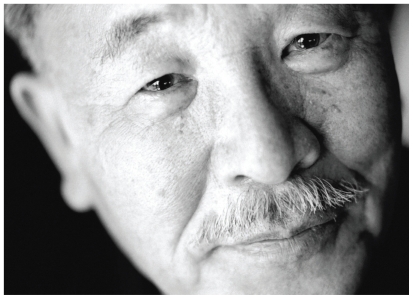# Organochlorines and Prostate Cancer in Japan: No Link in Men without Occupational Exposures

**DOI:** 10.1289/ehp.118-a216b

**Published:** 2010-05

**Authors:** Kris S. Freeman

**Affiliations:** **Kris S. Freeman** has written for *Encarta* encyclopedia, NIH, ABCNews.com, and the National Park Service. Her research on the credibility of online health information appeared in the June 2009 *IEEE Transactions on Professional Communication.*

Occupational exposure to organochlorine compounds during pesticide manufacturing or application has been associated with prostate cancer incidence. However, prostate cancer was not clearly associated with plasma levels of organochlorine compounds among Japanese men in the general population, according to results of a large-scale prospective study **[*EHP*** **118:659–665; Sawada et al.]**.

Organochlorines can act as endocrine disruptors. Studies in animals and humans have reported evidence of associations between significant environmental exposures and effects such as urogenital malformation in boys born to agricultural workers. Organochlorines were banned in the 1970s in Japan, where the current study was based. However, because these compounds persist in the environment, environmental exposures may still be affecting human health.

In a nested case–control study, the authors tracked the incidence of prostate cancer among 14,203 men aged 40–69 who were enrolled in a prospective study through the Japan Public Health Center. Participants responded to baseline health questionnaires and provided blood samples between 1990 and 1995; they were followed through 2005. The authors identified 201 participants who were diagnosed with prostate cancer during the period of the study, each of whom was matched with two controls from the study cohort. The baseline blood samples from these 603 men were analyzed for polychlorinated biphenyls (PCBs) and several organochlorine pesticides.

The authors found no statistically significant associations between blood levels of any organochlorine and prostate cancer. Contrary to expectations, men who developed cancer had lower blood levels of the pesticides hexachlorobenzene and β-hexachlorocyclohexane than men who did not develop cancer, though these inverse associations were not statistically significant.

Strengths of the study include the large number of participants and the use of biological samples collected many years before diagnosis. However, the authors acknowledge the small number of cases of prostate cancer may have limited their ability to detect associations with organochlorine exposures. Moreover, the length of followup may have been insufficient to fully detect incidence of prostate cancer, which is generally slow to develop.

## Figures and Tables

**Figure:**